# Neuroanatomy of melanocortin-4 receptor pathway in the mouse brain

**DOI:** 10.1515/biol-2020-0063

**Published:** 2020-08-13

**Authors:** Kun Wang, Wei Mao, Xiaoyu Zhang, Yufei Zhao, Kuikui Fan, Deng Pan, Haodong Liu, Penghui Li, Rihan Hai, Chenguang Du

**Affiliations:** Institute of Cereal and Oil Crops, Hebei Academy of Agriculture and Forestry Sciences, Shijiazhuang 050000, China; College of Veterinary Medicine, Inner Mongolia Agricultural University, Hohhot 010018, China; Vocational and Technical College, Inner Mongolia Agricultural University, Baotou 014109, China; Inner Mongolia Key Laboratory of Basic Veterinary Science, Hohhot 010018, China

**Keywords:** melanocortin-4 receptor, hypothalamus, immunofluorescence, Western blot, central nervous system

## Abstract

**Objective:**

Melanocortin-4 receptors (MC4Rs) are key regulators of energy homeostasis and adipose deposition in the central nervous system. Considering that MC4R expression regions and function-related research mainly focus on the paraventricular nucleus (PVN), little is known about their distribution throughout the mouse brain, although its messenger RNA distribution has been analyzed in the rat. Therefore, MC4R protein localization in mouse neurons was the focus of this study.

**Methods:**

MC4R protein distribution was assessed in mice through immunofluorescence and Western blotting.

**Results:**

MC4R was differentially expressed throughout the arcuate nucleus (ARC), nucleus of the solitary tract (NTS), raphe pallidus (RPa), medial cerebellar nucleus, intermediolateral nucleus, and brainstem. The highest MC4R protein levels were found in the ARC and ventromedial hypothalamic nucleus, while they were significantly lower in the parabrachial nucleus and NTS. The lowest MC4R protein levels were found in the PVN; there was no difference in the protein levels between the area postrema and RPa.

**Conclusions:**

These data provide a basic characterization of MC4R-expressing neurons and protein distribution in the mouse brain and may aid further research on its role in energy homeostasis.

## 1 Introduction

The melanocortin system in the central nervous system (CNS) plays an important role in regulating appetite and energy homeostasis [1]. As a potential mediator, the central melanocortin system regulates food intake [2], energy expenditure, and body weight via distinct projection patterns to melanocortin receptors (MCRs) in hypothalamic and extra-hypothalamic nuclei [3]. These effects are mediated mainly via G protein-coupled melanocortin-4 receptor (MC4R) activation and stimulated by the α melanocyte-stimulating hormone [4,5]. An MC4R deficiency results in obesity and many features of the metabolic syndrome, including insulin resistance, hyperinsulinemia, and increased visceral adiposity [6].

MC4Rs are expressed throughout various mammalian tissues; in particular, they are expressed in human skin, hair, eyes, and in the brain [7]. Over the last decade, functional MC4R research focused on the paraventricular nucleus (PVN) [8]. However, there are additional neuronal populations, in which MC4R activation controls the metabolic and cardiovascular functions. That also has been identified in other regions of the brain. In addition to the nucleus tractus solitarius (NTS), MC4R expression has also been located in the arcuate nucleus (ARC) and intermediolateral nucleus (IML) [9]. These findings suggest that MC4R expression in other neuronal populations, besides the PVN, is important in body weight regulation and may be involved in altering energy expenditure as well as food intake.

MC4R-green fluorescent protein (GFP) transgenic mice have recently been used to identify the MC4R distribution. However, through this approach, the recognition of MC4R expression is based on the MC4R-GFP specific fluorescent signal; this, along with a high cost, made this approach unsuited for public laboratories. Moreover, although MC4R-expressing PVN neurons promote satiety by projecting to and activating neurons in the NTS [10], the MC4R distribution pattern in the CNS has not been explored in detail. Furthermore, neuroanatomical MC4R studies have been limited by the availability of the MC4R-specific antibodies for the brain, whereas few studies have shown messenger RNA levels.

Exploring MC4R expression in regions other than the PVN will assist the research on energy homeostasis regulation. Accordingly, this study investigates the expression pattern of MC4R neurons and protein levels in the CNS using specific antibody labeling.

## 2 Materials and methods

### 2.1 Animal husbandry

C57/BL6j wild-type male mice (WT, 8–9 weeks old) were obtained from Charles River Laboratories (Beijing, China). The mice (*n* = 5) were maintained in a normal environment on a 12 h light/dark cycle (lights on 06:00–18:00) with *ad libitum* access to food and water and were fed a standard diet.


**Ethical approval:** The research related to animal use has been complied with all the relevant national regulations and institutional policies for the care and use of animals, and has been approved by the Institutional Animal Care and Use Committee (IACUC) of the Inner Mongolia University, in compliance with NIH guidelines (Protocol number – SYCK-002, approved in July 2014).

### 2.2 Immunofluorescence

Animals were deeply anesthetized and transcardially perfused with ice-cold 0.9% saline, followed by 4% paraformaldehyde-borate fixative (pH 7.4) for 20 min. The mouse brains were removed, then postfixed in the same fixative overnight at 4°C, followed by cryoprotection in 30% sucrose solution at 4°C before being processed for immunocytochemistry. Brains (*n* = 3) were subsequently sliced as 30 µm sections, encompassing the hypothalamus, with a cryostat microtome (Slee MNT, Mainz, Germany).

The sections were collected into four serially ordered sets of sections in 0.01 M phosphate-buffered saline (PBS). After an initial blocking step (3% normal donkey serum in 0.01 M PBS containing 1% Triton X-100), the sections were incubated with primary antibodies F rabbit anti-MC4R (1:1,000, ab24233, Abcam) overnight at 4°C. Next, the sections were washed and incubated with Alexa Fluor 647 Donkey anti-Rabbit IgG (1:1,000, ab150075, Abcam) for 2 h at about 25°C. After washes, the sections were mounted on frosted gelatin-coated slide and air-dried. All immunofluorescence experiments used the free-floating method under 300 rpm wobbling with an Eppendorf ThermoMixer C. As a negative control, we used the same samples and the same protocol without primary antibody. Fluorescent neuronal cells were visualized and imaged using a Nikon C2 confocal microscope (Japan) with 4× and 10× objectives.

### 2.3 Western blot

With the remaining animals, those not used for immunofluorescence, brains were removed into ice-cold 0.9% saline and sliced into 200 µm sections (bregma: −4.84 to −7.48 mm) with a vibrating microtome. PVN, VMH, ARC, PBN, NTS, AP, and raphe pallidus (RPa) tissues (*n* = 2) were collected by capillary glass tube with an aperture of 2 mm under stereomicroscope.

Total protein was extracted for the expression of MC4R. Specifically, brain tissue sections were cut into small pieces and powdered with liquid nitrogen, placed into a homogenization solution containing ice-cold RIPA (2333s, Beijing, China) and protease inhibitors (complete EDTA-free protease inhibitor cocktail; Roche Diagnostics, Alameda, CA), and then briefly sonicated (15 s/time, 90 V, 3 times). Protein concentrations were determined by BCA protein assay (Thermo Scientific, MA, USA), and approximately 30 µg of total protein was electrophoresed through a 12% running and a 5% stacking SDS-PAGE and wet transferred to a nitrocellulose transfer membrane (66485, Pall). Membranes were blocked with 5% nonfat dry milk and then incubated with primary antibodies and the corresponding secondary antibodies: rabbit anti-MC4R (1:1,000, ab24233, Abcam) and rabbit anti-GAPDH (1:10,000, ab181602, Abcam) overnight at 4°C. The membranes were subsequently washed followed by incubation with the corresponding IRDye® 800LT Goat anti-Rabbit (1:10,000; 926-32211, Odyssey) for 1 h at about 25°C. Detection and quantification were performed using an Infrared Imaging System (Odyssey; LI-COR Biosciences). Band intensities were determined using the median background method. The MC4R levels were normalized to GAPDH.

### 2.4 Statistics

Data analysis was done using Prism 7.0 software (GraphPad, San Diego, CA, USA). Data are presented as means ± standard errors of the means. Comparisons between the expression levels of MC4R in different tissues were made by repeated-measures ANOVA. Results with *P* values less than 0.05 were considered statistically significant.

## 3 Results

### 3.1 MC4R expression in the DG-mo, CB, hypothalamus, and MY

**Figure 1:**
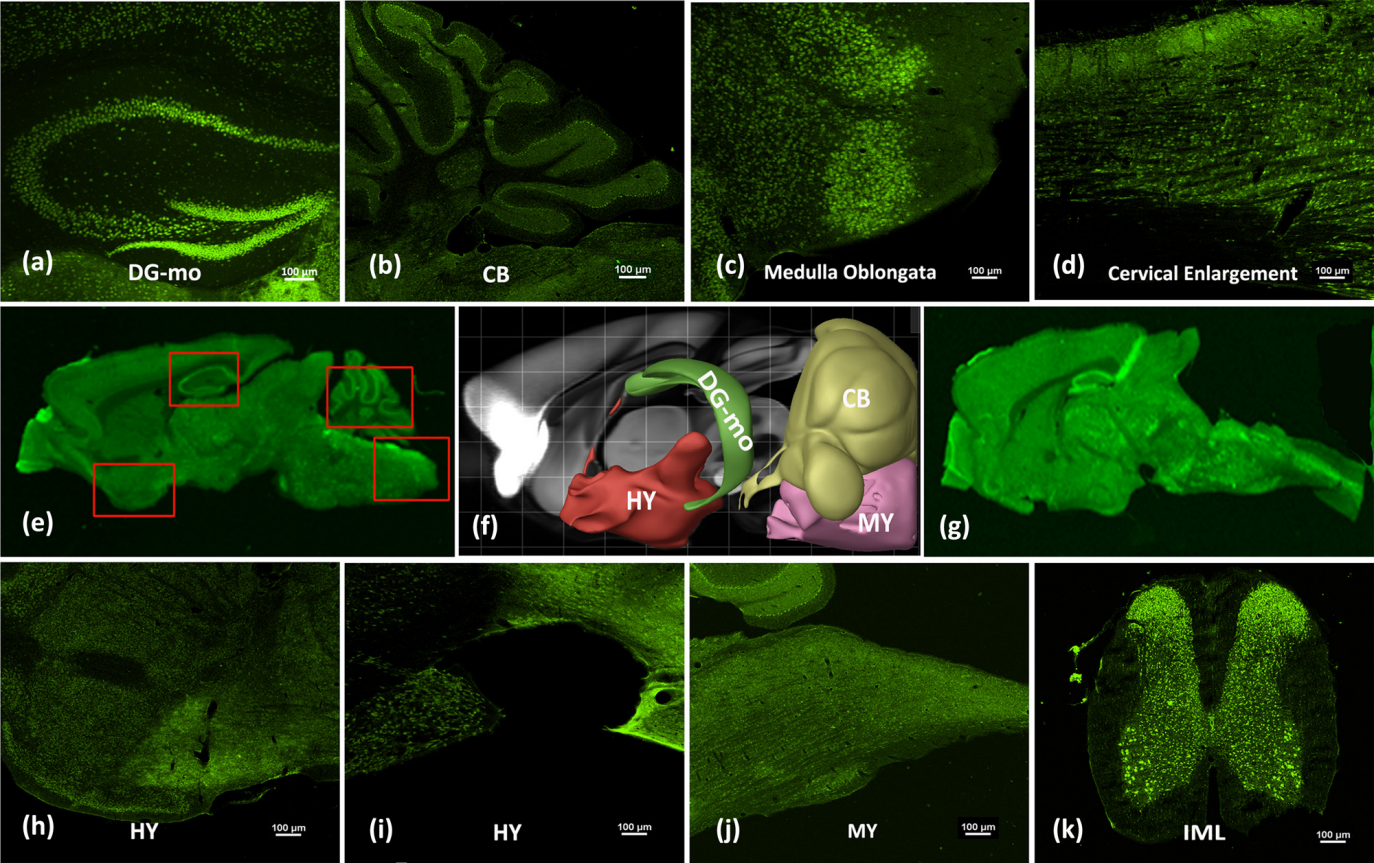
MC4R expression in the mouse CNS. Representative images of sagittal mouse brain sections showing MC4R (green) expression by immunofluorescence. (a) MC4R signal-labeled neurons were detected in the DG-mo. (b) The CB showed MC4R-positive cells. (c) MC4R labeling in the medulla oblongata is shown. (d) MC4R labeling in the cervical enlargement is shown. (e) Sagittal left-to-right views of the whole brain are shown. (f) The Brain Map–Brain Explorer 2 from the Allen Institute for Brain Science. (g) Negative control. (h and i) MC4R expression in the hypothalamus is shown. (j) MC4R expression in the MY is shown. (k) MC4R expression in the IML is shown. Abbreviations: CB, cerebellum; DG-mo, dentate gyrus molecular layer; IML, intermediolateral nucleus; MC4R, melanocortin-4 receptor; MY, medulla. Scale bars: 100 µm.

In sagittal brain sections, a very high density of MC4R-labeled neurons was detected in the dentate gyrus molecular layer (DG-mo; Figure 1a). Other areas of the brain showed moderate signal intensity. The cerebellum (CB; Figure 1b) also showed a very strong MC4R expression. Intense MC4R immunoreactivity was found along the medulla (Figure 1c), an area influencing blood pressure and respiration. Within the region next to the medulla, homogenous MC4R staining was found on the cervical enlargement region (Figure 1d) and hypothalamus (Figure 1h and i). Labeling was also detected within the rhombencephalon, specifically the medulla oblongata (MY; Figure 1j). As expected, we did not observe any staining with the negative control (Figure 1g).

Compared with other hindbrain regions, an abundant MC4R-positive signal was observed in the unique architecture of the IML (Figure 1k) that transmits via parasympathetic preganglionic fibers. MC4R signaling neurons were observed in both the medulla and spinal cord regions. Overall, projections of MC4R-expressing neurons exited the CNS, suggesting that MC4R may be essential in regulating processes of CNS.

### 3.2 MC4R expression in the MT, VMH, and ARC

**Figure 2:**
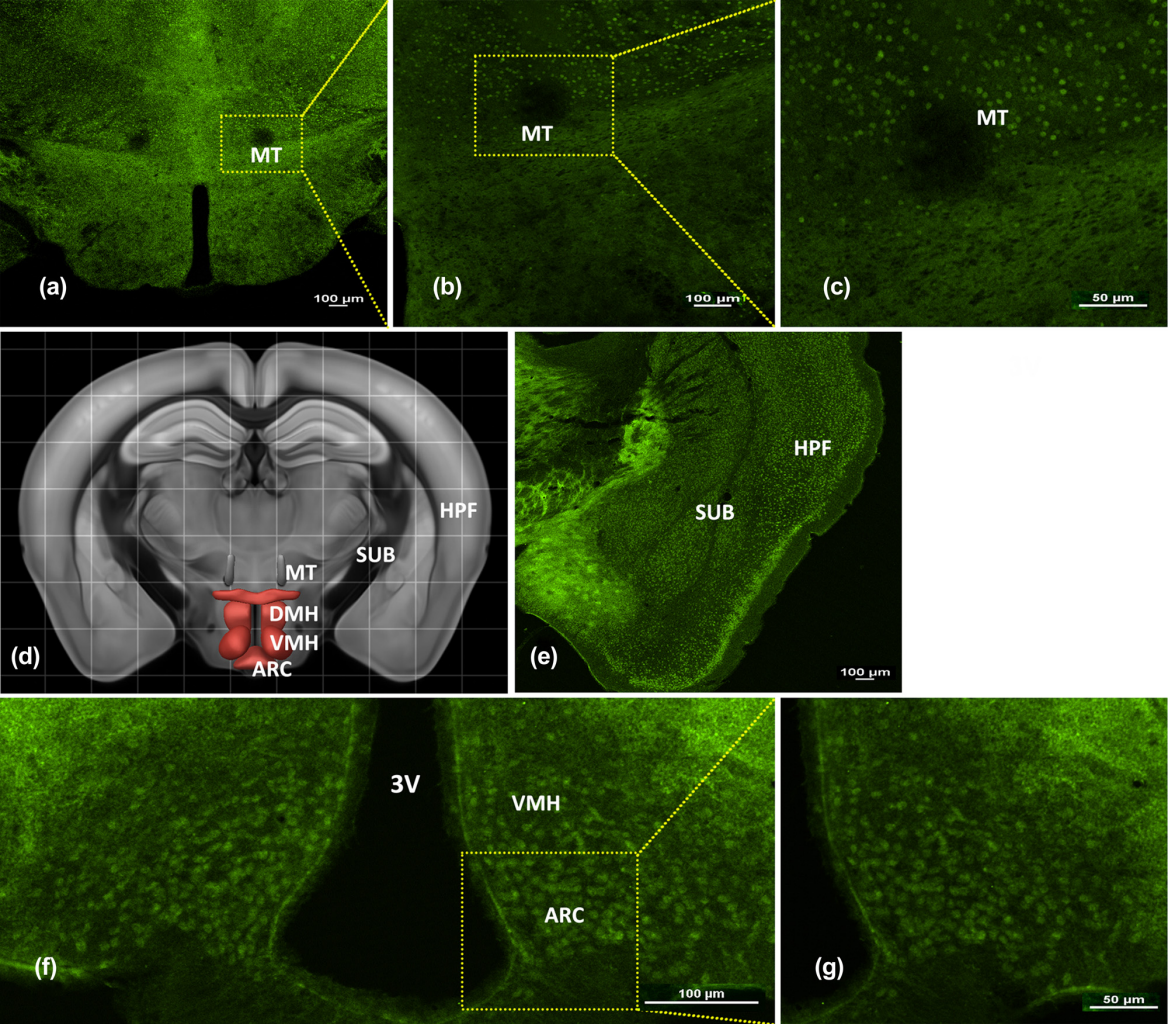
MC4R expression in the mouse forebrain. A picture collage of the representative coronal mouse brain sections showing the MC4R distribution within forebrain regions. (a) MC4R expression in the MT is shown. (d) The Brain Map–Brain Explorer 2 from the Allen Institute for Brain Science. (e) MC4R expression in the SUB and HPF is shown. (f) MC4R expression in ARC of the hypothalamus is shown. (b, c and g) Higher magnification of the left images is shown. Abbreviations: ARC, arcuate nucleus; DMH, dorsomedial hypothalamic; HPF, hippocampal formation; MC4R, melanocortin-4 receptor; MT, medial terminal nucleus of the accessory optic tract; SUB, subiculum; VMH, ventromedial hypothalamic nucleus; 3 V, third ventricle. Scale bars: 50 or 100 µm.

Positive MC4R staining was observed in coronal sections, as expected. Strong MC4R immunoreactivity was detected in the medial terminal nucleus layer (Figure 2a–c), and MC4R was homogenously expressed around the third ventricle (3V) throughout all areas of the forebrain. In the subiculum, stellate-like distributions of MC4R immunoreactivity were observed throughout, suggesting that these nerve cells expressed MC4R. Similarly, MC4R staining was intense around the hippocampal formation (Figure 2e); immunopositive neurons were largely confined to ARC areas (Figure 2f and g). Throughout the hypothalamus, a structure central to neuroendocrine function, MC4R immunoreactivity was evident in the ventromedial hypothalamic nucleus (VMH) and ARC.

### 3.3 MC4R expression in the NTS, PB, and RPa

**Figure 3:**
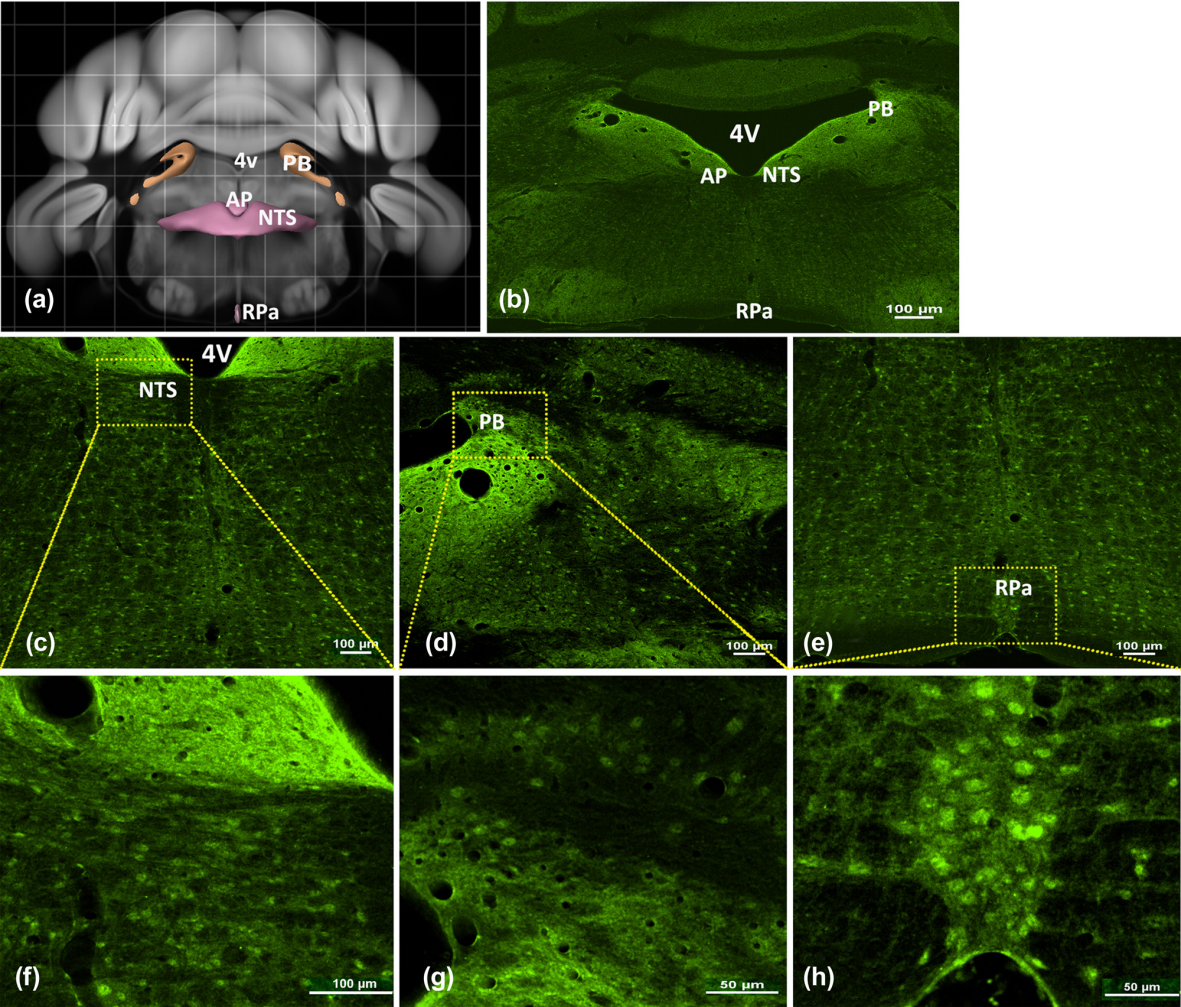
MC4R expression in the mouse hindbrain. Representative micrographs showing MC4R expression in the mouse brain in hindbrain regions. (a) The Brain Map–Brain Explorer 2 from the Allen Institute for Brain Science. (b) The coronal–caudal to rostral view of the whole brain. (c) MC4R expression in the NTS is shown. (d) MC4R expression in the PB is shown. (e) MC4R expression in the RPa is shown. (f–h) Higher magnification of the upper images. Abbreviations: AP, area postrema; MC4R, melanocortin-4 receptor; NTS, nucleus of the solitary tract; PB, parabrachial nucleus; RPa, raphe pallidus nucleus; 4V, fourth ventricle. Scale bar: 50 or 100 µm.

Within rhombencephalon regions, MC4R expression was prominent in cells surrounding the fourth ventricle (4V) (Figure 3b). MC4R labeling was detected in the NTS (Figure 3c and f) with robust expression surrounding the 4 V. Notably, intense nerve cell MC4R immunoreactivity was seen throughout the parabrachial nucleus (PBN) (Figure 3d and g). Furthermore, compared with other hindbrain regions, a large distribution was observed in the RPa (Figure 3e and h). MC4R staining was not intense in the area postrema, but was evident in a region likely involved in energy intake control. The present data show intense MC4R immunoreactivity throughout the hindbrain.

### 3.4 MC4R protein expression levels in key nuclei of energy metabolism

**Figure 4:**
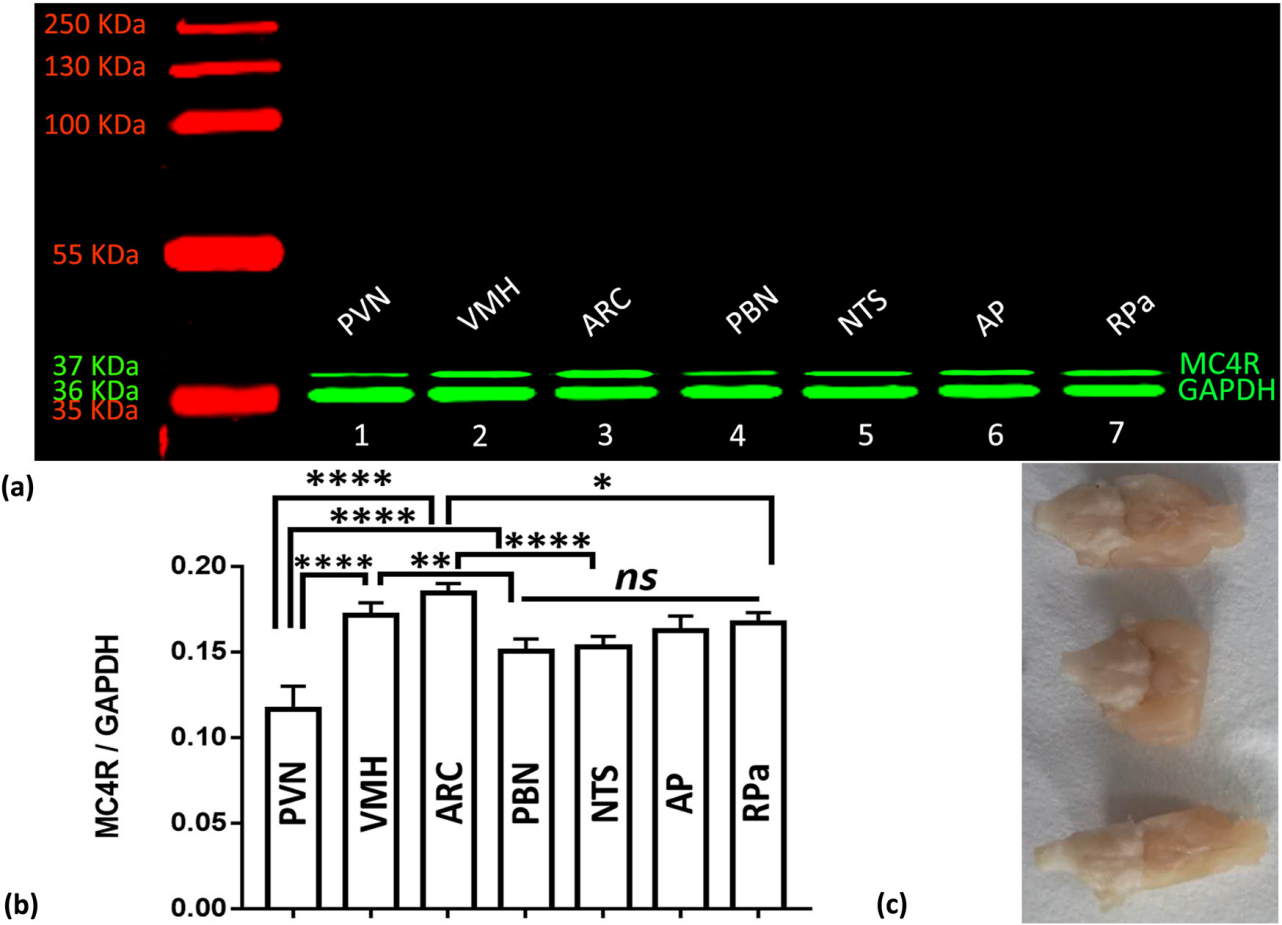
MC4R protein expression in the mouse brain. Western blot analysis results of various brain regions. (a) A representative blot of MC4R monomers (37 kDa, upper band) and GAPDH (36 kDa, lower band) proteins in the PVN, VMH, ARC, PBN, NTS, AP, and RPa of the mouse brain is shown. (b) MC4R band intensities were scanned, quantified, and normalized to the corresponding GAPDH level. (c) Materials of brain for Western blotting. *indicates *P* < 0.05; **indicates *P* < 0.01; ***indicates *P* < 0.001; ****indicates *P* < 0.0001; and ns indicates no significance when compared with the brain regions. Abbreviations: ARC, arcuate nucleus; AP, area postrema; GAPDH, glyceraldehyde-3-phosphate dehydrogenase; MC4R, melanocortin-4 receptor; NTS, nucleus of the solitary tract; PBN, parabrachial nucleus; PVN, paraventricular nucleus; RPa, raphe pallidus nucleus; VMH, ventromedial hypothalamus.

MC4R immunoreactivity was detected in the ARC (Figure 4a) and dorsal root ganglion in the same manner as previously reported with MC4R-GFP [11,12]. Protein was isolated from the PVN, VMH, ARC, PBN, NTS, AP, and RPa and separated by SDS-PAGE (Figure 4c). The highest MC4R protein expression was observed in the ARC and VMH, with a significantly lower expression in the PBN and NTS; the lowest amount was observed in the PVN. There was no difference in protein expression between the AP and RPa.

A significant difference was observed between the MC4R protein levels in the ARC and those in the AP, RPa (*P* = 0.0037 and 0.0030, respectively), and PVN (*P* < 0.0001). The VMH, compared with the PBN (*P* = 0.0070) and NTS (*P* = 0.0239), demonstrated significantly higher MC4R levels than the PVN (*P* < 0.0001) and the AP and RPa (*P* = 0.9778). No significant differences were observed between the NTS and PBN (*P* = 0.9987), or AP and RPa (*P* = 0.9778, Figure 4b).

## 4 Discussion

Here, we describe that the MC4Rs are expressed in the VMH and ARC region, as previously reported [13], in addition to many other CNS regions. Several extra-hypothalamic areas showed considerable MC4R expression, including the hippocampus, cerebral cortex, CB, DG-mo, MY, as well as several brainstem and spinal cord nuclei. Overall, this study provides a foundation for exploring the neurochemical phenotype of central melanocortinergic neurons.

MC4R expression is located in the PVN [14] and ARC [3] regions in mice, regulating energy metabolism in different ways [15]. Specifically, MC4R is a critical factor for maintaining hypothalamic appetite regulation [16]. Furthermore, the MC4R expression pattern also suggests a specialized role in energy homeostasis regulation. For instance, MC4R knockout mice lack the ability to properly maintain energy homeostasis and, for this reason, may be more prone to obesity [11].

The hypothalamus is a critical structure for controlling food intake and energy expenditure [17], implicating the melanocortin system as a signaling pathway that drives energy homeostasis. The melanocortin system is involved in amylin-induced food intake suppression and thermogenesis activation in both the hypothalamus and brown adipose tissue (BAT) via modulation of acetyl-CoA carboxylase phosphorylation and uncoupling protein-1 expression [3]. The current findings agree with previous results suggesting regional hypothalamic MC4R expression, with the highest MC4R levels near the 3V and posterior brain regions [18]. Furthermore, marked MC4R downregulation was observed in an animal model of obesity [11], in agreement with the phenotype of the MC4R knockout mice [12], suggesting a role for MC4R in energy metabolism. These data, together with recent evidence indicating that MC4R modulates BAT thermogenic activity during energy expenditure in the hypothalamus, suggest that MC4R is essential for energy metabolism [3,10]. The evolution of endocrine and neuronal MCR-mediated circuits has been shaped by the coevolution of the MCR gene family, the proopiomelanocortin (POMC) gene, and genes that code for polypeptides that interact with the receptors as reverse agonists (i.e., agouti gene-related protein [AGRP]). AGRP [19], neuropeptide Y [20], and POMC act on MC4R [21] and are involved in mediating energy homeostasis. Hence, the central melanocortin system is implicated in energy homeostasis with many gene interactions.

Here, Western blotting and immunohistochemistry results elucidated the MC4R expression pattern throughout the mouse brain, providing a comparatively comprehensive neuroanatomical study of MC4R localization and protein levels. Notably, a region-specific MC4R expression pattern was demonstrated. Comparable to other studies, the MC4R expression was highly centralized with intense ARC immunoreactivity. Similarly, immunoreactivity was found in the PVN [15], terminating around the 3V. The PVN is an important regulatory center of neuroendocrine activity and the sympathetic nerve drive, while the hypothalamic ARC is involved in anterior pituitary endocrine function regulation, many metabolic processes, and complex behaviors. Robust staining indicated a marked MC4R expression in the hippocampus, ARC, PBN, NTS, RPa, and AP; expression was also found in the VMH, DMH, and IML, but very little immunoreactivity was found medial to the PVN [22] and brainstem. These patterns agree with a previous study [3,23]. Importantly, MC4R expression was particularly high in the ARC, a structure hypothesized to be important in metabolism and other functions related to energy consumption [24]. The heterogeneous MC4R expression pattern throughout the brain suggests various functional roles for MC4R, in addition to its role in homeostasis similar to findings in the MC4R-GFP mouse reports [23,25]. Our study agrees with and expands on previously published results, providing detailed data on the distribution of MC4R in the CNS.

## 5 Conclusion

This study comprehensively characterized MC4R distribution and, using direct labeling with specific antibodies, found brain areas with high expression. MC4Rs were densely expressed in metabolism-processing regions surrounding the 3V and in brain regions associated with the hypothalamus, including the ventricles, midbrain and hindbrain regions. Regionally, MC4R protein expression was highest in the ARC, where it was strongly expressed in the dense nerve network throughout the brain. Furthermore, MC4Rs demonstrated region-specific results within individual structures. Thus, by investigating the local relationship between MC4R expression and specific anatomical regions, a detailed distribution and morphological characterization of MC4R protein in the brain were provided that could aid further research into its function.
